# Factors associated with empowerment after participating in a supported osteoarthritis self-management program: An explorative study

**DOI:** 10.1016/j.ocarto.2024.100464

**Published:** 2024-03-26

**Authors:** Karin Sturesdotter Åkesson, Eva Ekvall Hansson, Teresa Pawlikowska, Anne Sundén, Kjerstin Stigmar, Eva Ageberg

**Affiliations:** a Department of Health Sciences, Health Science Centre, Box 157, SE-221 00, Lund, Sweden; b Health Professions Education Centre, RCSI University of Medicine and Health Sciences, Dublin, Ireland

**Keywords:** Osteoarthritis, Empowerment, Patient education, Physical therapy, Exercise therapy

## Abstract

**Objective:**

To explore factors associated with change in empowerment in patients that have participated in a 3-month Supported Osteoarthritis Self-Management Program (SOASP). Further, to evaluate empowerment in the longer term.

**Design:**

An explorative analysis including patients from a cohort study conducted in primary healthcare in Sweden was performed. Univariable linear regression models were performed to assess associations between demographics and patient-reported outcome measures (explanatory factors), respectively, and change in empowerment from baseline to 3-month follow-up (outcome variable). Long-term follow-up of empowerment was at 9 months.

**Results:**

Self-reported increase in enablement at the 3-month follow-up was associated with a greater improvement in empowerment (B ​= ​0.041, 95% CI (0.011, 0.07), p ​= ​0.008). Living alone was associated with less improvement in empowerment (B ​= ​−0.278, 95% CI (−0.469, −0.086), p ​= ​0.005) compared to living together. Physical exercise >120 ​min per week at baseline was associated with less improvement in empowerment (B ​= ​−0.293, 95% CI (−0.583, −0.004), p ​= ​0.047) compared to reporting no exercise at baseline. No other associations were observed (p ​> ​0.05). Empowerment improved from baseline to the 3-month follow-up (mean 0.20 (SD 0.5), p ​< ​0.001) but there was no change from baseline to the 9-month follow-up (mean 0.02 (SD 0.6), p ​= ​0.641).

**Conclusions:**

Self-reported increased enablement may lead to greater improvement in empowerment after SOASP. Greater efforts may be needed to support those that live alone, are physically active, and to sustain empowerment in the longer term after SOASP. More research is needed on empowerment to provide personalized support for patients with OA after SOASP.

## 1 Introduction

The World Health Organization (WHO) emphasizes the importance of self-management for patients with chronic diseases. i.e., osteoarthritis (OA) [1]. Given that OA prevalence is expected to increase [2] and health care resources are limited [3,4], patients with OA will need to take on a greater responsibility for self-managing their disease in the coming years [5]. Patient education is an important intervention to increase self-management [1] and is included in the recommended first-line treatment, along with exercise and weight loss (if needed), for patients with OA [[6], [7], [8]].

According to national guidelines in Sweden, patients with OA should be offered first-line treatment in primary health care through a Supported Osteoarthritis Self-Management Program (SOASP) that combines patient education and exercise [9]. The SOASP has been described in detail [9]. In brief, it often starts with two to three educational group sessions, led by a physiotherapist (PT), where patients learn about OA i.e., the diagnosis, etiology, risk factors and current treatment. After the educational sessions, patients are offered an individually adapted exercise program to practice either at home or in a group led by a physiotherapist. Patients diagnosed with OA are to be offered to participate in SOASP preferably as soon as possible after diagnosis [4,9]. The main exclusion criterion for participation in SOASP is having symptoms and/or pain due to causes other than OA [9].

The overall aim of SOASP is to increase patient empowerment and self-management [9,10]. However, there are no guidelines, frameworks, or theories recommended for clinicians to rely on in how to educate, empower and enable patients with OA to self-manage their disease [11]. Both empowerment and enablement are important concepts in relation to patient education and self-management. Patient empowerment is defined as “a process to gain control over decisions that affect one's health and personal life” [1]. Patient enablement, as measured by the Patient Enablement Instrument, is described as a patient's ability to understand and cope with their illness after a health care consultation [12,13], is viewed as a subset of patient empowerment [14].

While pain and function are routinely evaluated through a national quality registry, the Swedish Osteoarthritis Registry [10], patient empowerment is so far not evaluated in the registry. In previous observational prospective studies in real-world clinical settings [15,16] we reported that empowerment increased among patients after participating in a 3-month SOASP [15]. However, it is unclear whether this improvement will be maintained in the longer term. In another study [16], we observed that empowerment was not associated with change in health-related quality of life (HRQoL) after a SOASP. Empowerment has been studied in other contexts and for other diagnoses, such as congenital heart disease [17], rheumatoid arthritis [18], diabetes [19], and arthritis [20]. A longitudinal observational study reports that low levels of empowerment are associated with worse pain-related factors, worse physical function, lower HRQoL, and lower physical activity levels [18]. Also, a randomized controlled trial reports that empowering interventions improve quality of life among patients with poorly controlled type 2 diabetes [19].

Identifying factors associated with change in patient empowerment after an intervention may enable health care personnel to provide greater personalized support for patients with OA to self-manage their disease. Research on empowerment in relation to OA is in its early stages [11], and to our knowledge there are no studies on factors associated with change in empowerment in patients with OA after participating in SOASP. Therefore, the main aim of this study was to explore factors associated with change in empowerment in patients that have participated in a Supported Osteoarthritis Self-Management Program (SOASP). A secondary aim was to evaluate empowerment after SOASP in the longer term.

## 2 Methods

### 2.1 Design and setting

This is an explorative analysis including patients from a cohort study [15,16] that was conducted in primary health care in Sweden. The study was approved by the Regional Ethical Review Board in Lund, Sweden (2015/918). The study was reported in accordance with the Strengthening the Reporting of Observational Studies in Epidemiology (STROBE) (Checklist provided Additional file 1) [21] and was retrospectively registered on 28/11/2016 on ClinicalTrials.gov (NCT 02974036).

### 2.2 Participants

Data was collected from 143 patients who participated in a 3-month SOASP in Sweden between 2016 and 2018 [15]. Inclusion criteria in the study were patients with hip and/or knee OA understanding Swedish and participating in the SOASP in primary health care. Exclusion criteria were not understanding Swedish. All patients provided written consent to participate in the study. In the current study, we included patients with data for empowerment at all time-points, i.e., prior to the participation in the SOASP (baseline), and at the 3-month (n ​= ​115) and the 9-month (n ​= ​116) follow-ups.

### 2.3 Data collection

**Fig. 1:**
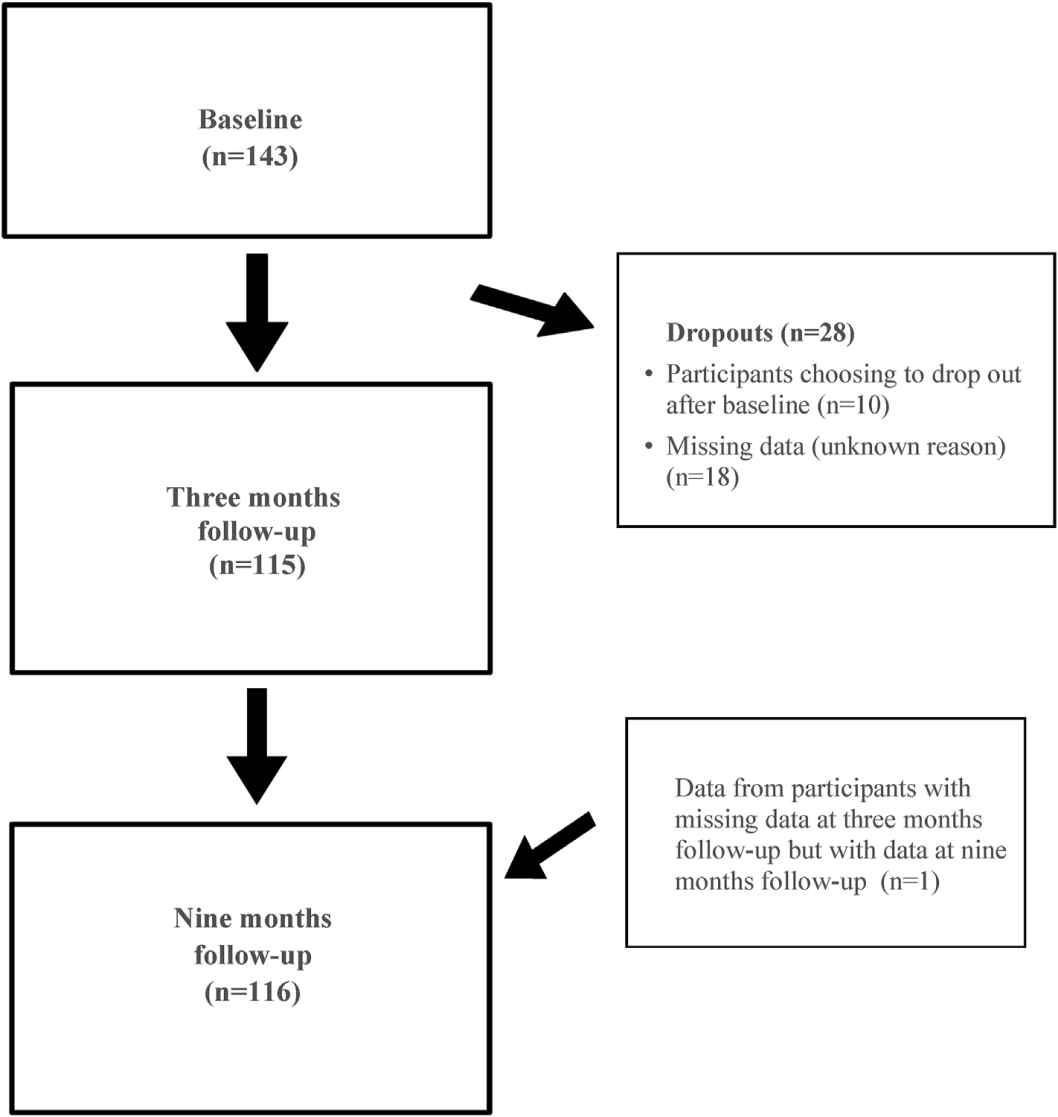
Flowchart for collection of data.

Data was collected consecutively by the PT responsible for the SOASP at the seven participating primary health care centers in two regions in the south of Sweden at baseline and at three months follow-up. The first author collected data at nine months follow-up through a postal questionnaire. A flowchart for collection of data is presented in Fig. 1 (Fig. 1).

Patients reported the following demographics at baseline: age, sex, Body Mass Index (BMI), most affected joint, highest level of education, civil status, and work situation. Generic and condition-specific patient-reported outcome measures (PROMs) were used to assess patients' health status. These included patients’ self-reported pain, physical exercise, everyday exercise, and health-related quality of life (HRQoL) at baseline, empowerment at baseline and at the 3- and 9-month follow-ups, and enablement at the 3-month follow-up after SOASP.

### 2.4 Outcome variable

Empowerment was measured using the Swedish version of the Swedish Rheumatic Disease Empowerment Scale (SWE-RES-23) [22]. The SWE-RES-23 is a condition-specific instrument developed from the Swedish Diabetes Empowerment Scale [22,23]. The phrase “rheumatic disease” in the original SWE-RES-23 was replaced with “osteoarthritis” in our study to make it clearer for the participating OA patients. When developed, the SWE-RES-23 was tested by patients with rheumatic disease including patients with OA [22]. The instrument consists of 23 questions with five answer alternatives ranging from “strongly disagree” (scored 1) to “strongly agree” (scored 5) [22]. As an example, the first three questions in the SWE-RES-23 are: *In terms of how I take care of my osteoarthritis, I …:* 1) *… know what parts of self-care that causes dissatisfaction,* 2) *… know what parts of self-care that I am ready to change,* and 3) *… know what parts of self-care that I am not ready to change.* The total score is calculated by summing the score of all questions and dividing the sum by 23, resulting in a total score between 1 and 5, where a higher score indicates higher empowerment. The SWE-RES-23 has shown acceptable construct validity and internal consistency reliability [22]. Change in empowerment from baseline to 3-month follow-up was used as outcome variable.

### 2.5 Explanatory variables

In total, 12 explanatory variables were investigated i.e., seven demographic variables (age, sex, BMI, most affected joint, highest level of education, civil status, and work situation) and five PROMs (pain, physical exercise, everyday exercise, HRQoL, enablement). The baseline values were chosen for all the explanatory variables except for enablement.

#### 2.5.1 Demographic characteristics

Age was reported in years and sex as man or woman. BMI (kg/m^2^) was calculated using patient's reported height in meters (m) and weight in kilograms (kg). In addition, patients reported most affected joint (hip, knee and/or hand), highest level of education (elementary school, upper secondary school, or university), civil status (living together or living alone), and work situation (retired, working or studying, sick leave fulltime, sick leave part time or unemployed).

#### 2.5.2 Patient-reported outcome measures

Pain was reported using the 11-point Numerical Rating Scale, (NRS-11) from 0 (“no pain”) to 10 (“worst imaginable pain”) [24]. The instruction was to “rate your pain by indicating the square that best describes your pain on average in your most affected joint in the last week”.

The two questions about physical exercise and everyday exercise were asked as recommended by the Swedish National Board of Health and Welfare [25]. Both questions refer to an ordinary week. For physical exercise, the question is: “How much time do you devote to physical activity that makes you short of breath, for example running, keep-fit exercises, or ball games?” The six categorical answer alternatives are no time, less than 30 ​min, 30–60 ​min, 60–90 ​min, 90–120 ​min, and >120 ​min. The question on everyday exercise is: “How much time do you devote to everyday physical activity such as walking, bicycling, or gardening? Include all activities lasting 10 ​min at a time.” The seven categorical answer alternatives are no time, less than 30 ​min, 30–60 ​min, 60–90 ​min, 90–150 ​min, 150–300 ​min, and >300 ​min [26]. The questions have shown stronger concurrent validity than open-ended questions when validated against accelerometer [26].

Health-related quality of life was measured using the Swedish version of the EQ-5D [27], a generic instrument [28,29]. Both the descriptive part (EQ-5D-5L) and the visual analogue scale (EQ VAS) were used in the analysis. The EQ-5D has been carefully described [27,30].

Enablement was measured using the Swedish version of the Patient Enablement Instrument (PEI) [12,13,31,32]. It is a generic instrument that consists of six questions with four answer alternatives: much better (scored 2), better (scored 1), same or less (scored 0), not applicable (scored 0), resulting in a total score between 0 and 12 [12,13,31], where a higher score indicates higher enablement. The PEI is a patient driven outcome measure, i.e., based on the patients’ own perception of change in enablement, developed to be answered after a consultation [13], thus, there is no baseline data reported for the PEI [13]. Therefore, data collected at the 3-month follow-up after SOASP was used for enablement. The Swedish version of the PEI has shown high internal consistency and moderate to good reliability [32] and fair content validity, construct validity, and internal consistency [33].

### 2.6 Patient partner

We engaged a patient partner (PP) in the study process. In the previous studies [15,16], our PP gave input on the research process from planning the study to interpreting the results. In the current study, the PP gave feedback on our interpretation of the results. Involving a PP in the process enhanced the patient perspective, and reporting followed the Guidance for reporting of patient and public involvement (GRIPP 2) (Checklist provided Additional file 2) [34].

### 2.7 Statistical analysis

The main characteristics and the study variables were described using frequencies (n) and percentages (%) for categorical variables and mean and standard deviation (SD) for continuous variables.

The change in empowerment from baseline to the 3- and 9-month follow-ups, respectively, were reported as mean and standard deviations, and the change from baseline to 9-months was analyzed for long-term result of empowerment.

Univariable linear regression models were performed to assess whether any explanatory factors were associated with the outcome variable, i.e., change in empowerment from baseline to 3-month follow-up. Explanatory variables were data on demographics at baseline (age, sex, BMI, most affected joint, highest level of education, civil status, and work situation) and PROMs (pain, physical exercise, everyday exercise, and HRQoL at baseline and enablement at 3-month follow-up). Where applicable, we consistently selected the category with the most frequent responses as the reference in the univariable linear regression models. The effect (B) with 95 % confidence interval (CI) and significance level α ​= ​0.05 were reported.

A sample size calculation for our previous study [15] was performed. The sample size calculation showed that 110 participants were needed. SAS Enterprise Guide 6.1 for Windows (SAS Institute Inc., Cary, NC, USA) was used for sample size calculation. Before initiating our study, we decided to collect data from a minimum of 140 participants to compensate for potential missing data. In practice, this resulted in data from 143 participants at baseline being collected due to the organization of the SOASP and data collection in this study followed usual clinical practice. No imputation was made for missing values [35]. Analysis was performed with SPSS version 28 (IBM corporation, New York, USA).

## 3 Results

Descriptive data on demographics and PROMs for the study cohort are presented in Table 1.

**Table 1:** Descriptive data on demographics and patient-reported outcome measures (PROMs) for the study cohort at baseline (n ​= ​143).

Demographics	Study cohort
Age (years)	
mean (SD)	65.9 (9.3)
min-max	40–90
Sex % (n)	
men	22 (32)
women	78 (111)
BMI mean (SD)	28.6 (5.4)
Most affected joint % (n)	
knee	72.1 (101)
hip	25.7 (36)
hand	2.1 (3)
missing data	2.7 (3)
Education % (n)	
elementary school	19.6 (28)
upper secondary school	30.8 (44)
university	46.9 (67)
missing data	2.8 (4)
Civil status % (n)	
living alone	35 (50)
living together	62.9 (90)
missing data	2.1 (3)
Work situation % (n)	
retired	62.2 (89)
work or study	26.6 (38)
Sick leave fulltime	4.9 (7)
Sick leave part time	3.5 (5)
Unemployed^a^	0.7 (1)
Missing data	2.1 (3)
**PROMs**	
PEI^b^	
mean (SD)	5.9 (3.1)
Pain^c^	
mean (SD)	5.4 (2.4)
Physical exercise % (n)	
no exercise	25.9 (37)
<30 ​min.	18.2 (26)
30–60 ​min.	20.3 (29)
60–90 ​min.	10.5 (15)
90–120 ​min.	6.3 (9)
>120 ​min.	16.8 (24)
Everyday exercise % (n)	
no exercise	2.1 (3)
<30 ​min.	9.1 (13)
30–60 ​min.	15.4 (22)
60–90 ​min.	18.9 (27)
90–150 ​min.	18.9 (27)
150–300 ​min.	21.0 (30)
>300 ​min.	12.6 (18)
EQ-5D-5L^d^	
mean (SD)	0.810 (0.125)
EQ VAS^e^	
mean (SD)	68.7 (19.3)
SWE-RES-23^f^	
mean (SD)	3.7 (0.6)

a Not included in analyses.

b PEI= Patient Enablement Instrument, measuring enablement at 3 months follow-up.

c Pain measured with the Numerical Rating Scale (NRS) at baseline.

d EQ-5D-5L: descriptive part of EQ-5D, measuring health-related quality of life at baseline.

e EQ VAS: visual analogue scale part of EQ-5D, measuring health-related quality of life at baseline.

f SWE-RES-23 measuring empowerment at baseline.

Empowerment (SWE-RES-23) improved from baseline to the 3-month follow-up (mean 0.2, SD 0.5, CI (0.10, 0.27), p ​< ​0.001) but not from baseline to the 9-month follow-up (mean 0.2, SD 0.6, CI (0.08, 0.13), p ​= ​0.641).

The univariable analyses are provided in Table 2. Self-reported increase in enablement (PEI) at the 3-month follow-up was associated with a greater improvement in empowerment (B ​= ​0.041, 95% CI (0.011, 0.07), p ​= ​0.008). Living alone was associated with less improvement in empowerment (B ​= ​−0.278, 95% CI (−0.469, −0.086), p ​= ​0.005) compared to living together. Physical exercise >120 ​min per week at baseline was associated with less improvement in empowerment (B ​= ​−0.293, 95% CI (−0.583, −0.004), p ​= ​0.047) compared to reporting no exercise at baseline. No other associations were observed between the outcome variable and the explanatory factors (p ​> ​0.05).

**Table 2:** Univariable linear regression for association between patient characteristics, and patient-reported outcome measures respectively and change in empowerment measured with SWE-RES-23 from baseline to 3 months follow-up (n ​= ​115).

Variables	Models		
	B	95% CI	*p*-value^h^
**Patient characteristics**			
Age	−0.001	−0.011; 0.009	0.791
Sex^a^	−0.002	−0.227; 0.224	0.987
BMI^b^	−0.006	−0.024; 0.012	0.523
Most affected joint			
Knee as reference			
Hip	−0.129	−0.345; 0.088	0.240
Education			
Elementary school as reference			
Upper secondary school	0.049	−0.222; 0.320	0.719
University	0.134	−0.118; 0.386	0.295
Civil status^c^	−0.278	−0.469; −0.086	0.005
Work situation			
Retired as reference			
Work or study	0.052	−0.168; 0.272	0.640
Sick leave fulltime	0.128	−0.317; 0.574	0.570
Sick leave part time	−0.244	−0.766; 0.277	0.355
**PROMs**			
PEI^d^ at 3 months	0.041	0.011; 0.070	0.008
Pain^e^ at baseline	−0.003	−0.042; 0.036	0.880
Physical exercise at baseline			
No exercise as reference			
<30 ​min.	0.031	−0.252; 0.314	0.828
30–60 ​min	0.095	−0.179; 0.369	0.495
60–90 ​min.	0.124	−0.214; 0.462	0.468
90–120 ​min.	0.096	−0.314; 0.507	0.643
>120 ​min.	−0.293	−0.583; −0.004	0.047
Everyday exercise at baseline			
150–300 ​min as reference			
No exercise	0.051	−0.616; 0.719	0.879
<30 ​min.	−0.159	−525; 0.207	0.391
30–60 ​min	0.253	−0.057; 0.562	0.108
60–90 ​min.	−0.079	−0.371; 0.213	0.592
90–150 ​min.	0.128	−0.164; 0.421	0.386
>300 ​min.	−0.210	−0.538; 119	0.208
EQ-5D-5L^f^ at baseline	0.213	−0.549; 0.975	0.580
EQ VAS^g^ at baseline	0.002	−0.003; 0.007	0.441

a Sex: 1 = woman, 0 = man.

b BMI = Body mass index.

c Civil status: 1 ​= ​living alone, 0 ​= ​living together.

d PEI= Patient Enablement Instrument, measuring enablement.

e Pain measured with Numerical Rating Scale (NRS).

f EQ-5D-5L: descriptive part of EQ-5D, measuring health-related quality of life.

g EQ VAS: visual analogue scale part of EQ-5D, measuring health-related quality of life.

h Significance level α ​= ​0.05.

## 4 Discussion

To our knowledge, this study is the first to report associations between self-reported patient empowerment and patients’ demographic characteristics and PROMs, respectively, after a Supported Osteoarthritis Self-Management Program. We found that self-reported increase in enablement at 3-month follow-up was associated with a greater improvement in empowerment from baseline to completing the SOASP (3-month follow-up). Moreover, reporting living alone and physical exercise >120 ​min per week at baseline were associated with less improvement in empowerment after SOASP. We found no further associations between the other explanatory factors and the outcome variable. Empowerment improved from baseline to the 3-month follow-up (p ​< ​0.001), but the improvement did not remain at the 9-month follow-up (p ​= ​0.641).

In this study, self-reported increase in enablement at the 3-month follow-up was associated with a greater improvement in empowerment. As enablement is described as “a preliminary competence, which can potentially progress into patient empowerment” [36], it could be that further emphasis on enablement during SOASP may improve empowerment.

Living alone was associated with less improvement in empowerment at the 3-month follow-up compared to living together. In a study on empowerment for patients with rheumatoid arthritis, patients reporting low empowerment were more often living alone [18]. To live alone is a risk factor for all-cause mortality [37] especially in older adults experiencing emotional loneliness [38]. During the SOASP, patients are supported by the PTs and by the group of peer patients participating in the SOASP at the same time. After 3 months, the SOASP is completed, and patients must manage on their own. Other studies have concluded that booster sessions after SOASP may be needed to sustain achieved outcomes in pain [39], HRQoL [40] and physical exercise [[40], [41], [42]], and future studies need to explore whether booster sessions affect (sustained) empowerment. Future studies may also explore other factors that might be associated with patient empowerment after SOASP, such as support, emotional loneliness, and potential effects of interventions aimed to enhance peer support on empowerment.

Patients who reported physical exercise >120 ​min per week at baseline showed less improvement in empowerment compared to patients reporting no exercise at baseline. As physical exercise is included in SOASP, it is likely that patients who reported no exercise at baseline had more room for improvement in empowerment than patients who reported a higher amount of physical exercise at baseline. The possibility of a potential ceiling effect on the results may be considered [43].

No other demographic characteristics or PROMs were associated with empowerment in our study. In other studies, demographic characteristics, such as sex and age, have shown to be associated with empowerment [18,20], however, results are ambiguous. In the study of the (Arthritis Foundation's) INSIGHTS program, lower empowerment was associated with being a man, older and/or with lower level of education [20]. In another study on patients with rheumatoid arthritis, lower empowerment was associated with being a woman and slightly older [18]. Patient-reported outcome measures of pain [18,44], level of physical activity [18,44] and HRQoL [18,19,44] have been associated with empowerment in other studies [18,19,44]. Patients with rheumatoid arthritis that reported lower empowerment, also reported more pain, and lower HRQoL and level of physical activity [18]. In a study of a 6-week empowerment-based intervention for patients with type 2 diabetes, the level of empowerment and quality of life increased at a 3-month follow-up [19]. Thus, the inconclusive results from various studies [[17], [18], [19], [20],^44^] indicate that more research is needed to optimize support to OA patients. Moreover, the scarce existing research of empowerment and its associations with OA, makes meaningful comparisons with other research studies limited, and emphasizes the exploratory nature of the current study.

In our study, the increase in empowerment at 3 months was not sustained at the 9-month follow-up. The improvement in empowerment in our study is equivalent to improvements in PROMs after SOASP reported in other studies [40,45,46] i.e., patient-reported physical exercise and HRQoL tend to increase in the short term (3-month follow-up) and decrease over the longer term (12-month follow-up) [40,46]. Whether the short-term increase of empowerment is due to participation in SOASP, or to other yet unknown factors, should be further investigated.

## 5 Strengths and limitations

Strengths of our study were that the response rate was high, with few missing data, and that data was collected in routine clinical practice, i.e., in the real-world setting. Moreover, involving a patient partner during the research process added to the patient perspective and validated the research question.

There are some limitations to acknowledge. First, we cannot exclude selection bias. Only Swedish speaking patients were included of which the majority reported rather good health status on several of the PROMs (HRQoL, everyday exercise, and empowerment) already at baseline, i.e., before participating in SOASP. Thus, the study cohort may not be representative of the general OA population in Sweden [15,16]. As patient participation in SOASP is voluntary, it could be that patients with more severe symptoms declined, or were not offered, to participate in SOASP, an issue that has been raised in other studies of SOASP [47]. As we have been told by the participating PTs, there are various reasons for dropouts such as some patients dropped out due to improvement and operation. However, it was not possible to keep a record of which of the above reasons applied to which patient given that the data was collected in a real-world setting. Second, the study cohort was too small to allow for any subgroup analysis, for example related to sex or joint.

## 6 Conclusion

In conclusion, our study provides new insights into factors that are associated with change in patient empowerment after SOASP. Self-reported increased enablement may lead to greater improvement in empowerment after SOASP. However, patients with higher amount of physical exercise at baseline may have less room for improvement in empowerment. Also, greater efforts may be needed to support those that live alone, and to sustain empowerment in the longer term after SOASP. Associations between empowerment and other factors need to be studied further to promote self-management and provide personalized support for patients with OA after SOASP.

## Author contributions

KSÅ and EA designed the current study, while KSÅ, EEH, TP, AS and KS contributed with the conception of the original study. KSÅ was responsible for acquisition of data, and KSÅ and EA for the analysis and interpretation of data. KSÅ drafted the article. EA revised early versions of the drafts critically for important intellectual content. EEH, TP, AS and KS revised later versions. All authors read and approved the final version to be submitted. KSÅ, karin.sturesdotter_akesson@med.lu.se, takes full responsibility for the integrity of the work, from inception to finished article.

## Role of the funding source

Open access funding provided by Lund University. This study was supported by the Swedish Rheumatism Fund (project no's: R-658001, R-750091, R-843831), the Stig Thune's Fund (project no's: 20181008, ST-201908), and the Greta and Johan Kocks's Foundation (project no's: 2019-10-22, 2020-10-19), and Governmental funding of clinical research within the National Health Services (NHS) (project no: 2018-Projekt0080 and 2022-Projekt0050, PI Eva Ageberg). The funding bodies did not take any part in the study design, collection, analysis, or interpretation of data, in writing the manuscript or the decision to submit the manuscript for publication.

## Data statement

The dataset generated and analyzed in this study is not publicly available due to the ethics approval and Swedish law.

The data used in this study contains sensitive information about the study participants and they did not provide consent for public data sharing. The current approval by the Regional Ethical Review Board in Lund, Sweden (2015/918) does not include data sharing. A minimal data set could be shared by request from a qualified academic investigator for the sole purpose of replicating the present study, provided the data transfer is in agreement with EU legislation on the general data protection regulation and approval by the Swedish Ethical Review Authority.

## Conflicts of interest

The authors declare that they have no competing interests.
