# Supplementary material for: Factors associated with empowerment after participating in a supported osteoarthritis self-management program: An explorative study
Source: Osteoarthr Cartil Open. 2024 Mar 26;6(2):100464. doi: 10.1016/j.ocarto.2024.100464 (PMC10998234; doi:10.1016/j.ocarto.2024.100464)
Supplement: Multimedia component 1 [file mmc1.docx]

**GRIPP2 short form**

| **Section and topic** | **Item** | **Reported on page No** |
| --- | --- | --- |
| 1: Aim | Report the aim of PPI in the study | 7 |
| 2: Methods | Provide a clear description of the methods used for PPI in the study | 7 |
| 3: Study results | Outcomes—Report the results of PPI in the study, including both positive and negative outcomes | 11 |
| 4: Discussion and conclusions | Outcomes—Comment on the extent to which PPI influenced the study overall. Describe positive and negative effects | 11 |
| 5: Reflections/critical perspective | Comment critically on the study, reflecting on the things that went well and those that did not, so others can learn from this experience | 11-12 |
